# Changing patterns of opioid agonist therapy prescribing in a network of specialised clinics providing care to people with opioid use disorder in Victoria, Australia, 2015 to 2023

**DOI:** 10.1111/dar.14049

**Published:** 2025-03-26

**Authors:** Joshua Dawe, Anna Lee Wilkinson, Michael Curtis, Jason Asselin, Charles Henderson, Eric Makoni, Paul Dietze, Margaret Hellard, Mark Stoové, the Australian Collaboration for Coordinated Enhanced Sentinel Surveillance of Blood Borne Viruses and Sexually Transmissible Infections (ACCESS)

**Affiliations:** ^1^ Disease Elimination Burnet Institute Melbourne Australia; ^2^ Global Epidemiology and Modelling Group University of Bristol Bristol UK; ^3^ School of Public Health and Preventive Medicine Monash University Melbourne Australia; ^4^ Melbourne School of Population and Global Health University of Melbourne Melbourne Australia; ^5^ National Drug Research Institute Curtin University Melbourne Australia; ^6^ NSW Users and AIDS Association Sydney Australia; ^7^ School of Health Research Menzies and Charles Darwin University Darwin Australia; ^8^ Department of Infectious Diseases Alfred Health and Monash University Melbourne Australia; ^9^ Doherty Institute and Melbourne School of Population and Global Health University of Melbourne Melbourne Australia; ^10^ Australian Research Centre in Sex, Health and Society La Trobe University Melbourne Australia

**Keywords:** buprenorphine, methadone, opiate substitution treatment, opioid agonist therapy, opioid dependence

## Abstract

**Introduction:**

Long‐acting injectable buprenorphine (LAIB) reduces the frequency of contact with opioid agonist therapy (OAT) service providers. Limited data exist on OAT prescribing in Australia after the introduction of subsidised LAIB prescribing in September 2019. This ecological study describes trends in OAT prescribing between 2015 and 2023 across a network of primary care services in Victoria, Australia.

**Methods:**

We utilised electronic medical records from 17 clinics in Victoria that provide services to people with opioid dependence to describe OAT prescribing patterns. We described the annual number and type (methadone, buprenorphine, LAIB) of OAT prescriptions issued, individuals prescribed, and individuals initiating OAT. Interrupted time series assessed changes in quarterly OAT prescribing following the introduction of LAIB.

**Results:**

Between 2015 and 2023, the average annual number of OAT prescriptions issued, and the average number of recipients prescribed OAT were 47,648 and 6470, respectively. Between 2020 and 2023, the proportion of individuals initiating on LAIB increased from 7% (73/1078) to 31% (357/1146). There was increasing quarterly OAT prescribing before the introduction of LAIB, after which methadone and buprenorphine prescribing declined by 2.6% (CR 0.974; 95% CI 0.968–0.980) and 3.2% (CR 0.968; 95% CI 0.963–0.973), respectively. After being introduced, quarterly LAIB prescribing increased by 13.1% (CR 1.131; 95% CI 1.096–1.167).

**Discussion and Conclusions:**

We found substantial changes in OAT prescribing patterns in Victoria between 2015 and 2023, with shifts away from oral methadone and sublingual buprenorphine to LAIB. Alongside ongoing monitoring of prescribing patterns, future research should assess how LAIB impacts patient health and social outcomes.

## 1 INTRODUCTION

Opioid agonist therapy (OAT) is an effective treatment for opioid dependence [1]. Monitoring OAT prescribing patterns is important for understanding service demand and informing evidence‐based policies and guidelines to support high‐quality patient care. Historically, OAT prescribing in Australia has predominantly involved oral methadone and sublingual buprenorphine [2]. In September 2019, long‐acting injectable buprenorphine (LAIB) was listed on the Australian Pharmaceutical Benefits Scheme alongside methadone and sublingual buprenorphine as a subsidised OAT formulation, with access expanded to non‐specialist settings in April 2020 [3]. Individuals with a valid Australian Medicare card are therefore only required to pay up to AU$31.60 per prescription (or AU$7.70 with a concession card), with the Australian Government subsidising the remaining cost [4]. Unlike the majority of OAT formulations in Australia, which require medical or pharmacy supervised dosing [2], LAIB is slow‐release buprenorphine in weekly or monthly formulations [5].

Facilitating access to appropriate treatment for people living with opioid dependence is a public health priority; an estimated 15,106 people in Victoria are currently being prescribed OAT [6]. For some, LAIB may be preferred due to the reduced frequency of clinical and pharmacy visits and associated impost on their lives and out‐of‐pocket expenses [7, 8, 9, 10]. While there is preliminary evidence suggesting that LAIB uptake is high among people with opioid dependence [7, 11, 12, 13], there remains limited real‐world data describing patterns of OAT prescribing in the context of widespread availability of LAIB [14, 15]. Data from sentinel surveillance networks are useful to monitor population‐level health‐care utilisation and evaluate changes in policy and practice.

Using electronic medical record data from primary care services that provide health care for people with opioid dependence, we aim to describe trends in OAT prescribing between 2015 and 2023, before and after the introduction of subsidised LAIB in September 2019.

## 2 METHODS

### 2.1 Setting

The Australian Collaboration for Coordinated Enhanced Sentinel Surveillance of Blood Borne Viruses and Sexually Transmissible Infections (ACCESS, accessproject.org.au) is a sentinel surveillance system which monitors trends in testing, diagnosis, and management of blood borne viruses and sexually transmissible infections among priority populations. A detailed explanation of ACCESS has been previously published [16]. Briefly, non‐identified electronic medical records are routinely extracted from participating clinical and laboratory services, and a unique hash code is created to link patient records between and within services. Ethical approval, including waiver of consent, for the ACCESS project was granted by the Human Research Ethics Committee of Alfred Hospital in Melbourne (248/17).

In this ecological study, data were drawn from 17 Victorian primary care services participating in ACCESS. Sites provided health services specific to people who inject drugs, including sterile needle and syringe dispensing, onsite hepatitis C testing and treatment, and OAT prescribing, alongside general health care. Included individuals were aged 16 years and over and were prescribed OAT at least once between 1 January 2015 and 31 December 2023. Data were electronic records of OAT prescription, including the date of prescription and OAT formulation; there were three outcomes for this study: LAIB prescriptions, oral methadone prescriptions and sublingual buprenorphine prescriptions.

### 2.2 Analyses

Analyses were performed using Stata version 18.0 for Windows (StataCorp, Texas, USA). Analysis code and simulated data for demonstration purposes can be accessed via https://github.com/JoshuaDaweUoB/LAIB_ITSA.

### 2.3 Descriptive analysis

Data were aggregated to annual numbers of: (i) OAT prescriptions; (ii) unique individuals; and (iii) individuals initiating OAT within the 17 ACCESS network clinics, stratified by OAT medication. Initiation of OAT was the first recorded OAT prescription within the ACCESS network for everyone. Individuals whose first prescription was before 1 January 2015 were not included in the analysis of OAT initiation. Individuals were counted once within each calendar year. Individuals with multiple OAT prescriptions within a calendar year were counted once but were categorised as ‘multiple OAT’.

### 2.4 Outcome one: Quarterly LAIB prescribing

To estimate the relationship between time (quarters) and count of LAIB prescriptions (outcome one), we fit a generalised linear model (Poisson), with an identity link function and robust standard errors. LAIB prescriptions between 1 October 2019 and 31 December 2023 were used; the exposure variable of time (continuous variable; 1 to 17) was therefore 17 quarters (1 October 2019–31 December 2023). The model provides a β coefficient and 95% confidence interval (CI), exponentiated to a count ratio, and interpreted as the relative average change in the mean count of LAIB prescriptions as time increases.

### 2.5 Outcomes two and three: Quarterly methadone and buprenorphine prescribing

To identify changes in overall, oral methadone and sublingual buprenorphine prescribing following the introduction of subsidised LAIB in September 2019, we conducted an interrupted time series as described in Xiao et al. [17]. The interruption was quarter four (1 October–31 December) of 2019, to correspond with the introduction of subsidised LAIB in September 2019. We included 36 quarters for all three OAT formulations, 19 before the introduction of LAIB (1 January 2015–31 September 2019), and 17 after (1 October 2019–31 December 2023). Analyses were conducted using step change generalised linear models (Poisson) with robust standard errors (for autocorrelation), with time (continuous variable; 1 to 36 quarters) and a dichotomous variable (0,1) representing if quarters were before or after the availability of subsidised LAIB. Model predicted OAT prescriptions issued if subsidised LAIB had not been introduced, based on the estimated coefficient for the pre‐LAIB period ($\beta_{1}$) provided the counterfactual. We derived indicator variables for Q1 (January–March) and Q4 (October–December), which were included as independent variables to assess for seasonal trends, and found no evidence of seasonality in the data. These variables were therefore not included in the final model. The models estimated the below coefficients, exponentiated and interpreted as:

$\beta_{1_{\text{time}}}$: The quarterly trend in the count of prescribing before the introduction of LAIB (pre‐slope).

$\beta_{2_{\text{LAIB}}}$: The immediate relative change in the count of quarterly prescribing after the introduction of subsidised LAIB (step‐change).

$\beta_{3_{\text{time}-\text{time}\left(i\right)\times \text{LAIB}}}$: The difference in the trend in the count of prescribing following the introduction of subsidised LAIB compared to pre the introduction (interaction term).

The trend in prescribing after the introduction of subsidised LAIB was estimated by ${\beta_{1}}_{\text{time}}+\beta_{3_{\text{time}-\text{time}\left(i\right)\times \text{LAIB}}}$.

## 3 RESULTS

Between 1 January 2015 and 31 December 2023, the average annual number of OAT prescriptions was 47,648 (SD 2804), and the average annual number of individuals prescribed OAT was 6470 (SD 567) (Table 1). Approximately two thirds (265,946/428,836; 62%) of OAT prescriptions over the 9 years were for methadone. The highest number of OAT prescriptions was in 2020 (51,727), and the lowest number of OAT prescriptions was in 2015 (44,212). In 2023, LAIB accounted for one‐quarter of all OAT prescriptions (10,952/45,893; 23.9%).

**TABLE 1:** Annual OAT prescribing within ACCESS by OAT type, Victoria, Australia, 1 January 2015–31 December 2023.

OAT prescribing	Total, *n* (%)	2015, *n* (%)	2016, *n* (%)	2017, *n* (%)	2018, *n* (%)	2019, *n* (%)	2020, *n* (%)	2021, *n* (%)	2022, *n* (%)	2023, *n* (%)
Overall prescribing										
Total prescriptions	428,835	44,212	44,644	45,428	50,230	49,837	51,727	49,969	46,895	45,893
Methadone	265,946 (62)	29,524 (67)	29,310 (66)	29,211 (64)	31,958 (64)	32,151 (65)	33,457 (65)	30,713 (61)	25,756 (55)	23,866 (52)
Buprenorphine	135,272 (32)	14,688 (33)	15,334 (34)	16,217 (36)	18,272 (36)	17,588 (35)	16,103 (31)	14,005 (28)	11,990 (26)	11,075 (24)
LAIB	27,617 (6)	0 (0)	0 (0)	0 (0)	0 (0)	98 (0)	2167 (4)	5251 (11)	9149 (20)	10,952 (24)
Client prescribing										
Clients prescribed	14,628	5614	5609	6092	6686	6595	6818	6768	6845	7205
Methadone	5176 (35)	3454 (62)	3356 (60)	3629 (60)	3857 (58)	3916 (59)	3912 (57)	3643 (54)	3327 (49)	3232 (45)
Buprenorphine	3912 (27)	1805 (32)	1886 (34)	2113 (35)	2307 (35)	2194 (33)	2004 (29)	1755 (26)	1610 (24)	1650 (23)
LAIB	639 (4)	0 (0)	0 (0)	0 (0)	0 (0)	3 (0)	139 (2)	493 (7)	942 (14)	1411 (20)
Multiple OAT^a^	4901 (34)	355 (6)	367 (7)	350 (6)	522 (8)	482 (7)	763 (11)	877 (13)	966 (14)	912 (13)
OAT initiation^b^										
Total	9389	1053	889	1241	1265	952	1078	853	912	1146
Methadone	3906 (42)	546 (52)	409 (46)	631 (51)	608 (48)	431 (45)	466 (43)	288 (34)	220 (24)	303 (26)
Buprenorphine	4681 (50)	507 (48)	480 (54)	610 (49)	657 (52)	517 (54)	539 (50)	446 (52)	446 (49)	486 (42)
LAIB	802 (9)	0 (0)	0 (0)	0 (0)	0 (0)	4 (0)	73 (7)	119 (14)	246 (27)	357 (31)

*Note*: Clients were counted once per year.

Abbreviations: ACCESS, Australian Collaboration for Coordinated Enhanced Sentinel Surveillance of blood borne viruses and sexually transmissible infections; LAIB, long‐acting injectable buprenorphine; OAT, opioid agonist therapy.

^a^ Multiple OAT indicates that a client received an OAT prescription for more than one OAT formulation within a calendar year, or across the entire observation period in the total column.

^b^ OAT initiation refers to the first recorded OAT prescription for an individual at any of the primary care health services within the ACCESS network.

An average of 1043 (SD 152) individuals initiated OAT each year between 2015 and 2023. Between 2015 and 2019, approximately half of the individuals initiated OAT on either buprenorphine or methadone. Following the introduction of LAIB, the proportion of individuals initiating OAT on methadone decreased from 45% in 2019 to 26% in 2023. In 2023, one‐third of individuals initiating OAT were prescribed LAIB (357/1146; 31.2%) (Table 1).

**FIGURE 1:**
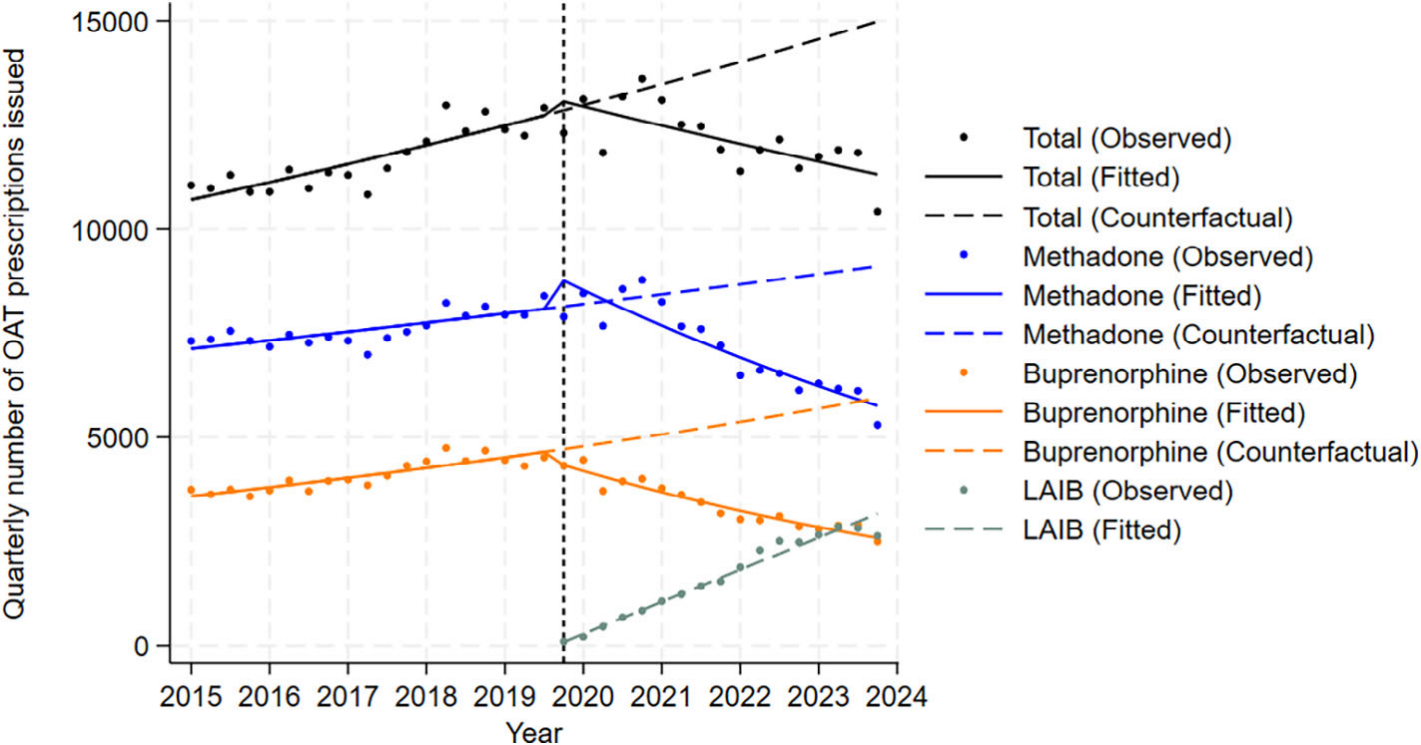
Observed, predicted and counterfactual predictions of the number of OAT prescriptions issued per quarter within ACCESS network before and after introduction of LAIB, by OAT formulation, Victoria, Australia, 1 January 2015–31 December 2023. Dashed vertical line corresponds with introduction of LAIB. An interrupted time series analysis was used for overall, methadone, and buprenorphine prescribing. A Poisson regression model was used for LAIB prescribing. Total OAT prescribing includes methadone, buprenorphine, and LAIB prescribing. ACCESS, Australian Collaboration for Coordinated Enhanced Sentinel Surveillance of blood borne viruses and sexually transmissible infections; LAIB, long‐acting injectable buprenorphine; OAT, opioid agonist therapy.

The quarterly number of prescriptions of both methadone and buprenorphine was increasing before the introduction of LAIB, at an average of 0.7% (count ratio [CR] 1.007; 95% CI 1.005, 1.009) and 1.4% (CR 1.014; 95% CI 1.011, 1.018) prescriptions per quarter, respectively (Table 2). The introduction of LAIB was associated with an immediate 8.3% decrease in buprenorphine prescribing (CR 0.917; 95% CI 0.862, 0.976), and an immediate 7.6% increase in methadone prescribing (CR 1.076; 95% CI 1.000, 1.158). Both methadone and buprenorphine had decreasing post‐LAIB trends, with prescribing decreasing by 2.6% (CR 0.974; 95% CI 0.968, 0.980) and 3.2% (CR 0.968; 95% CI 0.963, 0.973) on average per quarter, respectively (Table 2). Following its introduction, LAIB prescribing increased by an average of 13.1% prescriptions (CR 1.131; 95% CI 1.096, 1.167) per quarter. Visual inspection of Figure 1 suggests a divergence in the fitted and counterfactual models under the counterfactual scenario of no subsidised LAIB.

**TABLE 2:** Quarterly changes in OAT prescribing trends in the ACCESS network before and after the introduction of LAIB in September 2019, Victoria, Australia, January 2015–December 2023.

	Pre‐LAIB quarterly OAT prescribing trends ($\beta_{1_{\text{time}}})$	Immediate change in OAT prescribing trends $\left(\beta_{2_{\text{LAIB}}}\right)$	Post‐LAIB quarterly OAT prescribing trends $\left(\beta_{1_{\text{time}}}+\beta_{3_{\text{time}\times \text{LAIB}}}\right)$	Difference between pre‐ and post‐LAIB quarterly prescribing trends $\left(\beta_{3_{\text{time}\times \text{LAIB}}}\right)$
	Count ratio (95% CI)	Count ratio (95% CI)	Count ratio (95% CI)	Count ratio (95% CI)
Overall prescribing	1.010 (1.007, 1.012)	1.016 (0.961, 1.075)	0.991 (0.986, 0.996)	0.982 (0.976, 0.987)
Methadone	1.007 (1.005, 1.009)	1.076 (1.000, 1.158)	0.974 (0.968, 0.980)	0.967 (0.961, 0.974)
Buprenorphine	1.014 (1.011, 1.018)	0.917 (0.862, 0.976)	0.968 (0.963, 0.973)	0.954 (0.949, 0.960)
LAIB^a^	–	–	1.131 (1.096, 1.167)	–

Abbreviations: ACCESS, Australian Collaboration for Coordinated Enhanced Sentinel Surveillance of blood borne viruses and sexually transmissible infections; CI, confidence interval; LAIB, long‐acting injectable buprenorphine; OAT, opioid agonist therapy.

^a^ Poisson regression model was used to estimate the trend in quarterly LAIB prescribing between 1 October 2019 and 31 December 2023.

## 4 DISCUSSION

Our study used electronic medical record data from a network of 17 primary care services in Victoria, Australia, to describe OAT prescribing trends prior to and following the availability of subsidised LAIB. We found declining trends in the prescribing of sublingual buprenorphine and oral methadone following the introduction of LAIB and increases in the prescribing of LAIB.

Like other recent Australian studies [6, 14, 18], we found rapid and sustained increases in LAIB prescribing following its subsidisation through the Australian Pharmaceutical Benefits Scheme, in addition to reductions in oral methadone and sublingual buprenorphine prescribing. This aligns with our finding that an increasing proportion of clients initiating OAT are prescribed LAIB, with one‐third of clients with no prior OAT prescribing history prescribed LAIB in 2023. Further, while the overall number of OAT prescriptions has declined since the introduction of LAIB in 2023, the number of clients who were prescribed OAT was higher than any other year of observation, and the numbers of clients initiating OAT were the highest since 2018. While we are unable to ascertain the reasons for these increases via sentinel surveillance data, they may be a result of the increase in OAT prescribing options and flexibility following the introduction of LAIB. Regardless, our findings indicate that the addition of LAIB as an additional OAT prescribing option was quickly implemented in primary care services that provide specialised care for people with opioid dependence, supporting its inclusion in the Australian Pharmaceutical Benefits Scheme.

Our study provides important insights into OAT prescribing trends in Victoria, Australia, underscoring how sentinel surveillance systems can be used to monitor and evaluate changing trends in OAT prescribing, thus informing future health policy and decision making. Our work also highlights the considerable volume of OAT prescribing taking place within primary care settings, with our analysis representing around half of Victoria's OAT recipients [6]. Increasing trends in the number of clients engaged suggest that this level of prescribing is likely to persist in the future. Our findings also indicate that an increasing proportion of OAT prescriptions are issued for LAIB, supporting recent evidence suggesting that LAIB prescribing is likely to become the most commonly prescribed OAT formulation in the near future [18]. Given these changes in prescribing patterns, OAT prescribers would benefit from ongoing enhanced and tailored support to ensure they are aware of the support needs of clients accessing LAIB, including appropriate induction and formulation transfer protocols [19].

This study has limitations. First, OAT prescribing that occurs outside of the ACCESS network is not available in our dataset. Consequently, OAT prescribing data outside the ACCESS network is not available. Therefore, individuals classified as having initiated OAT within the ACCESS network may have been previously prescribed OAT elsewhere, and some prospective prescribing may have been missed if clients changed prescribers or re‐initiated OAT at clinics outside the network. Second, while ACCESS aims to collect data that is representative of people from priority populations, most of the services included in our analysis provide specialised care for people with opioid use disorder. Our analysis may therefore not be representative of prescribing that occurs in non‐specialised services.

## AUTHOR CONTRIBUTIONS

**JD:** conceptualisation, methodology, software, formal analysis, data curation, writing of the original draft, review and editing, visualization. **ALW:** conceptualisation, methodology, software, formal analysis, data curation, writing of the original draft, review and editing. **MC:** conceptualisation, methodology, writing of the original draft, review and editing. **JA:** project administration, funding acquisition, review and editing. **CH:** review and editing. **EM:** review and editing. **PD:** review and editing. **MH:** review and editing, funding acquisition, supervision. **MS:** conceptualisation, methodology, investigation, writing of the original draft, review and editing of the manuscript, supervision, funding acquisition.

## FUNDING INFORMATION

ACCESS receives core funding from the Australian Department of Health and Aged Care.

## CONFLICT OF INTEREST STATEMENT

The authors declare no conflicts of interest.

## Data Availability

Research data are not shared.
